# Ecophysiology and phylogeny of new terricolous and epiphytic chlorolichens in a fog oasis of the Atacama Desert

**DOI:** 10.1002/mbo3.894

**Published:** 2019-07-05

**Authors:** Patrick Jung, Dina Emrich, Laura Briegel‐Williams, Michael Schermer, Lena Weber, Karen Baumann, Claudia Colesie, Philippe Clerc, Lukas W. Lehnert, Sebastian Achilles, Jörg Bendix, Burkhard Büdel

**Affiliations:** ^1^ Plant Ecology and Systematics University of Kaiserslautern Kaiserslautern Germany; ^2^ Faculty of Agricultural and Environmental Science University of Rostock Rostock Germany; ^3^ Edinburgh Global Change Institute, School of GeoSciences University of Edinburgh Edinburgh Scotland; ^4^ Conservatoire et Jardin botaniques de la Ville de Genève Chambésy Switzerland; ^5^ Department of Geography Ludwig-Maximilians-University Munich Munich Germany; ^6^ Faculty of Geography Philipps University of Marburg Marburg Germany

**Keywords:** gas exchange, ITS, lichen, lichenicolous fungi, rbcL, *Trebouxia*

## Abstract

The Atacama Desert is one of the driest and probably oldest deserts on Earth where only a few extremophile organisms are able to survive. This study investigated two terricolous and two epiphytic lichens from the fog oasis “Las Lomitas” within the National Park Pan de Azúcar which represents a refugium for a few vascular desert plants and many lichens that can thrive on fog and dew alone. Ecophysiological measurements and climate records were combined with molecular data of the mycobiont, their green algal photobionts and lichenicolous fungi to gain information about the ecology of lichens within the fog oasis. Phylogenetic and morphological investigations led to the identification and description of the new lichen species *Acarospora conafii* sp. nov. as well as the lichenicolous fungi that accompanied them and revealed the trebouxioid character of all lichen photobionts. Their photosynthetic responses were compared during natural scenarios such as reactivation by high air humidity and in situ fog events to elucidate the activation strategies of this lichen community. Epiphytic lichens showed photosynthetic activity that was rapidly induced by fog and high relative air humidity whereas terricolous lichens were only activated by fog.

## Graphical abstract

The Atacama Desert is one of the driest and probably oldest deserts on Earth where indigenous lichens are reported to grow. This article presents the phylogeny of two epiphytic and two terricolous lichens, their photobionts and fungal parasites and describes their eco‐physiological adaptations.

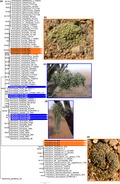

## 1 INTRODUCTION

The Atacama Desert in South America represents the driest place and the oldest desert on Earth and has experienced extreme hyperaridity for at least 3 million years (Hartley, Chong, Houston, & Mather, 2005). The desert stretches over more than 3,500 km along the western Pacific coastline from the region Trujillo in Peru near the Ecuadorian border (8°S) to La Serena in the centre of Chile (29°S). Its ancient temperate arid to hyper‐arid characteristics are caused by the Humboldt Current. In addition to surviving the low water availability, plant life must be adapted to extremely high intensities of photosynthetic active radiation (PAR), a high temperature amplitude and strong wind erosion. In contrast to the harsh desert environment, Las Lomitas, situated in close vicinity to the Pacific Ocean, represents a local fog oasis in the National Park Pan de Azúcar in the southern part of the Atacama Desert (Rundel, Dillon, & Palma, 1996). Here, fog together with dew and high relative air humidity are the main and frequent sources of water (Thompson, Palma, Knowles, & Holbrook, 2003). Fog water in this region is formed by upstreaming maritime air and as such it is the result of the combination of topography, seasonal wind field and trade inversion height (Borthagaray, Fuentes, & Marquet, 2010; Lehnert, Thies, et al., 2018; Muenchow et al., 2013). The occurrence of fog at Las Lomitas creates a narrow ecological niche for the establishment of at least some vascular plants with a very low coverage which is composed mainly of a few cacti and *Euphorbia* shrubs. Both length and intensity of fog events is highly variable resulting in a wide range of daily water inputs that can reach up to 16.6 mm precipitation available for lichens on a single day (Lehnert, Thies, et al., 2018). Lichens at Las Lomitas grow epiphytically on cacti and shrubs or as crustose patches on the ground (terricolous) (Baumann et al., 2018; Bernhard et al., 2018).

Lichens represent an obligate mutualistic ectosymbiosis between at least one eukaryotic green algal or cyanobacterial species (photobiont) and one fungal species (mycobiont). Recently, a number of ascomycete macrolichens have been found to consist of more than one mycobiont (Spribille et al., 2016), and for many other lichens, other associations as for example with lichenicolous fungi have been revealed. Although lichenicolous fungi are very common, little is known about their identity or the extent to which they can interact with their hosts (Lawrey & Diederich, 2003; Tuovinen et al., 2019). The poikilohydric character of lichens plays a crucial role during the survival of long periods of desiccation: lichens are inactive when dry but become rapidly reactivated when water is available (Green & Lange, 1995). Even high relative air humidity (>90%) has been shown to be sufficient for photosynthetic activity in a number of lichens with a green algal photobiont (chlorolichens) (Colesie, Green, Raggio, & Büdel, 2016; Lange, Kilian, & Ziegler, 1986; Raggio et al., 2017). In addition to high air humidity, epiphytic chlorolichens of the genera *Ramalina* and *Usnea* are known to thrive in the Atacama Desert in high abundance due to the capability to capture water from incoming fog. Fog interception should not be confused with condensation (Villegas, Tobón, & Breshears, 2008) as it is comparable to filter feeding by aquatic invertebrates (Larson, 1981; Stanton & Horn, 2013), and seems to be related to lichen growth form (Lange & Redon, 1983; Stanton, 2015). This was demonstrated for lichens from the coastal fog zone of the Namib Desert, where these lichens with the highest surface to volume ratio reached the highest photosynthesis rates and also made most effective use of different water sources (Lange, Meyer, Ullmann, & Zellner, 1991). Fog water capture by epiphytic fruticose lichens is so efficient, that it can result in dripping water, a frequently observed phenomenon in the Atacama Desert, which is known to influence the soil hydrology underneath the cacti (Stanton et al., 2014). However, it remains unclear to which extent terricolous lichens in the Atacama Desert can use different sources of water and how their morpho‐anatomical characteristics influence their ecophysiological pattern.

Morphological adaptations on various levels as for example growth form, anatomy but also photobiont diversity determine ecophysiological patterns (e.g. Colesie, Williams, & Büdel, 2017), which have rarely been investigated for lichens from the Atacama Desert (Lange & Redon, 1983). Since terricolous lichens have been recently reported to show a large range of adaptations and rapid responses to their environment (Green, Pintado, Raggio, & Sancho, 2018), the present study aims to compare and describe the ecophysiological patterns of the two most abundant terricolous and epiphytic lichens of an isolated lichen community. Further, the usage of various water sources and how thallus morpho‐anatomical and hydrological traits influence photosynthetic activity is investigated. Additionally, the mycobiont and photobiont of the examined lichens, as well as their specific lichenicolous fungi, are sequenced with a multi gene loci approach in order to elucidate their taxonomic identity.

## 2 MATERIAL AND METHODS

### 2.1 Study site

The National Park Pan de Azúcar is located between 25°53′ and 26°15′S, and 70°29′ and 70°40′W along the Pacific coast in Chile, in the southern part of the Atacama Desert. A narrow pediment close to the coast characterizes the local topography with a steep mountain ridge reaching altitudes up to 850 m a.s.l. that descends slightly towards the inland to altitudes between 700 and 400 m a.s.l. The study site “Las Lomitas” is located in a local fog oasis in the National Park Pan de Azúcar, close to the coastal crest of the first mountain ridge. The annual rainfall is less than 13 mm in average, but totals can be higher due to extreme precipitation events which occur occasionally in El Niño and El Niño‐like years when sea surface temperature anomalies off the coast are positive. An average temperature of 13°C during winter (July) and 20°C during summer (January) with daily maxima occasionally exceeding 26°C have been recorded (Rundel et al., 1996; Thompson et al., 2003). Relative air humidity under clear sky conditions is between 80% and 85% with fog affecting the coastal areas. Cacti such as *Eulychnia saint‐pieana* and shrubs of *Euphorbia lactiflua* dominate the vegetation at parts of Las Lomitas (Bernhard et al., 2018). Here, the cacti are highly occupied by epiphytic lichens (Stanton et al., 2014). Only a few crustose terricolous lichen species occur in this area of the Atacama Desert, forming a so‐called biological soil crust (BSC), which also includes cyanobacteria, free living green algae and fungi. They cover between 30% and 40% of the front ridge (Baumann et al., 2018; Lehnert, Jung, Obermeier, Büdel, & Bendix, 2018; Lehnert, Thies, et al., 2018). In August 2017, a high proportion of lichens were found to be associated with lichenicolous fungi, visible by blackish deformations or galls of the lichen thalli.

### 2.2 Climate stations

Three automatic weather stations were installed in March 2016 at Las Lomitas as described in Lehnert, Thies, et al. (2018). The stations were equipped with standard sensors measuring wind speed, wind direction, surface and air temperatures, relative humidity and precipitation as well as PAR. Fog water fluxes were measured using cylindrical fog collectors (“harp”‐type) and a dew balance at the ground level where BSCs (mainly crustose lichens) had been glued on. An analysis regarding fog and dew water fluxes was published in Lehnert, Thies, et al. (2018).

### 2.3 Sample collection

The two most abundant terricolous lichen species of the genus *Acarospora* and *Placidium* (both crustose growth form) (Bernhard et al., 2018), were sampled randomly along the ridge (25°59′03″S; 70°36′55″W; 764 m a.s.l.; Appendix Tables A1 and A2) by pressing eight sterile 9‐cm diameter Petri dishes 1 cm deep into the soil. Excess soil was removed with the Petri dish lid. Samples were air‐dried in the field immediately after collection. The two most abundant epiphytic lichens of the genus *Ramalina* (fruticose hairlichen) and *Everniopsis* (fruticose) (Stanton et al., 2014) were picked from cacti and *Euphorbia* bushes and stored in paper bags. Ten replicates of each lichen species were collected. The dry lichen samples were preserved at −20°C in plastic boxes until further processing. For this study, the samples were slowly defrosted before they were used for the laboratory analyses.

### 2.4 Isolation of photobionts

The algal partners of all lichen species were isolated from a clean thallus edge by removing small lichen pieces with a razor blade and carefully squeezing them between a microscope slide and cover slip to obtain a green suspension of algal cells and fungal hyphae. Under a binocular stereoscope, a group of algal cells was then transferred with a pipette to a petri dish with solidified Bold's Basal Medium as described for green algae in Baumann et al. (2018). After three to four weeks, visible algal colonies were streaked onto new plates for purification to obtain single‐cell based colonies. The isolates were used to capture microscopic images for comparison of the morphological features of the algae with the phylogenetic results.

### 2.5 Light microscopy

Thin sections of 20–25 µm thickness of the lichen thalli were prepared with a freezing microtome in order to maintain the inner structure of the lichen thalli. The samples were investigated with a light microscope with AxioVision software (Axioskop, Zeiss, Germany) under 630 magnification and oil immersion.

Cultured photobiont colonies were also investigated using light microscopy.

### 2.6 DNA extraction and amplification

Prior to DNA extraction, lichen fragments that were not affiliated with lichenicolous fungi were carefully washed with distilled water to remove epiphytic algae and adhesive soil under a binocular stereoscope. Specimens that were colonized by lichenicolous fungi were treated in the same way but here only blackish lichenicolous material was picked from the lichen thallus. Sample preparation was followed by DNA extraction using cetrimonium bromide method followed by phenol‐chloroform‐isoamyl alcohol purification adapted for lichens (Shivji, Rogers, & Stanhope, 1992). This resulted in genomic DNA from the mycobiont and its photobiont as well as the lichenicolous fungus. After DNA extraction the DNA was purified with the NucleoSpin^®^ Gel and PCR Clean‐up Kit (Macherey‐Nagel GmbH & Co. KG, Düren, Germany). The DNA was stored at −20°C until further processing.

Two gene loci were addressed for the mycobiont DNA (internal transcribed spacer [ITS], large subunit of RNA polymerase II [RPB1]), three gene loci for the green algal photobiont DNA (18S rDNA; 26S rDNA; large subunit of the plastid gene ribulose‐1,5‐biphosphate carboxylase/oxygenase [rbcL]) and one for the lichenicolous fungi (internal transcribed spacer [ITS]) in subsequent PCRs. Difficulties during sequencing of the mycobiont and lichenicolous fungi have been reported because universal DNA barcodes do not exist (Schoch et al., 2012). After testing several recent primer pairs and PCR conditions (Matheny, Liu, Ammirati, & Hall, 2002; Schmitt et al., 2009; Schoch et al., 2012; Westberg, Millanes, Knudsen, & Wedin, 2015; Williams et al., 2017; Zhao et al., 2017), the following final settings were used for the mycobiont: the primer pair ITS1f and LR3 (ITS) and gRPB1‐A and fRPB1‐C (RPB1) after Westberg et al. (2015) and Zhao et al. (2017) respectively. The lichenicolous fungi were inseparably connected to the lichen thallus and therefore always mixed with DNA of the mycobiont. Due to a high affinity for lichenicolous DNA the primer pair ITS1f and LR3 (ITS) were chosen to sequence the lichenicolous fungi. All fungal PCRs were conducted in a volume of 25 µl in illustra PuReTaq Ready‐To‐Go PCR bead tubes (GE Healthcare, UK), because common PCR methods were tested and found to be less successful. The green algal photobiont DNA was amplified with the primer pairs Al1500af (18S rDNA) and LR3 (26S rDNA) and rbcLf and rbcLr (rbcL) both after the conditions described in Williams et al. (2017).

The PCR products were purified with the NucleoSpin^®^ Gel and PCR Clean‐ up Kit (Macherey‐Nagel GmbH & Co. KG, Düren, Germany) and sent to SeqIT (Kaiserslautern) for Sanger sequencing.

All obtained sequences were submitted to the European Nucleotide Archive (ENA; Appendix Table A2).

### 2.7 Phylogeny

The sequences were BLASTed against the GenBank data base (http://blast.ncbi.nlm.nih.gov/Blast.cgi) in order to find the most similar sequences which were subsequently incorporated into the alignment. The ClustalW algorithm was applied for all alignments in Mega X (version 10.0.5, Kumar, Stecher, Li, Knyaz, & Tamura, 2018). One alignment for each gene region was prepared. Ambiguous regions were adjusted or removed manually allowing smaller final blocks and gap positions within the final blocks. *Xanthoria parietina* KJ027708.1 was included as root for the fungi and *Chlorella sorokiniana* KX495084.1 was included as root for the green algae trees. A Maximum Likelihood method with 500 bootstrap replications and the Kimura‐2‐parameter model (Kimura, 1980) was calculated for each alignment with Mega X as well as Bayesian phylogenetic analyses with Mr. Bayes 3.2.1 (Ronquist & Huelsenbeck, 2003). Both used gamma‐distributed rates.

The resulting phylogenetic trees were compared and as they did not differ significantly from each other the alignments were concatenated. One phylogenetic tree including two gene regions for the mycobiont, one tree including only the ITS gene region for the lichenicolous fungi and one tree including three gene regions for the photobionts were calculated as mentioned above and visualized using FigTree version 1.4.3. All alignments were deposited at dryad https://doi.org/10.5061/dryad.jc06126 and the publicly available sequences that were used to create the alignments can be seen in Appendix Tables A3, A4, A5.

### 2.8 Gas exchange

CO_2_ gas exchange measurements were conducted under controlled laboratory conditions using two minicuvette systems in parallel (CMS400 and GFS 3,000, Walz Company, Effeltrich, Germany). The CO_2_ gas exchange response in the light (net photosynthesis, NP) and in the dark (dark respiration, DR) was determined for five replicates of each terricolous lichen species and for five replicates of each epiphytic lichen species (Appendix Table A1) after Colesie, Green, Haferkamp, and Büdel (2014).

Before investigating the lichens’ NP response to different light conditions, the samples were submerged in water for 20 min at room temperature to guarantee water saturation. After removing them from the water they were shaken to remove excess water and placed into the cuvette and exposed to increasing light intensities (0; 15; 25; 50; 75; 100; 150; 300; 500; 1,000; 1,500; 2000 µmol photons m^−2^ s^−1^) at 17°C with ambient CO_2_ concentrations. Each measuring cycle started in the dark and lasted for 45 min until NP under the highest light intensity was reported. These cycles were repeated until the samples were completely dry. The light saturation point (LSP) was defined as the photosynthetic photon flux density at 90% of maximum NP. The light compensation point (LCP) was calculated as that light intensity at which NP compensates respiration.

Photosynthetic reactivation from high relative air humidity alone was tested by placing completely desiccated lichen samples in the gas exchange cuvette, exposed to an air stream of 90% and 95% relative air humidity, respectively at 17°C. The samples were kept in the dark for 45 min during which one measurement point was reported, representing the DR. Afterwards the light was switched on with an intensity of 800 µmol photons m^2^ s^−1^ for 15 min to obtain an NP value at the end of the interval. This light‐dark cycle was repeated for 24 hr.

To determine the optimal thallus water content for net photosynthesis, the samples were submerged in water for 20 min at room temperature to guarantee saturation directly after the experiment where they were exposed to 24 hr of high relative air humidity. After this, they were placed back into the cuvette at 800 μmol photons m^−2^ s^−1^, incident light, ambient CO_2_ concentrations and 17°C, which represents the realistic conditions found in the Atacama Desert (Bernhard et al., 2018). The CO_2_ exchange of the samples was recorded in alternating intervals in the dark to obtain DR values and afterwards during the given light intensity for NP values until the sample was completely desiccated and showed no further response. After each DR and NP measurement, the samples were weighed to report the weight loss by evaporating water during desiccation. After the measurement, the lichens were dried at 65°C for 3 days in a drying oven (Heraeus Instruments T6P, Thermo Fisher Scientific Inc.) and weighed to obtain their dry weight. The thallus water content during the measurements could then together with the dry weight of the sample be calculated as equivalent of mm precipitation.

After completion of all experiments the CO_2_ exchange of the samples was related to their chlorophyll content, chlorophyll_a+b_ was extracted after the method described by Ronen and Galun (1984).

### 2.9 Chlorophyll fluorescence

PAM fluorometry (pulse amplitude modulation) is based on the supply of weak, modulated light pulses (the measuring light), which allow chlorophyll fluorescence to be monitored without inducing photosynthesis. This method can be used to calculate the quantum yield of photosystem II (Y(II)) as a rapid response to different environmental conditions such as reactivation by fog. The quantum yield is measured under ambient light and calculated as Y(II) = (Fm′ − Ft)/Fm′, with Ft being the continuous chlorophyll fluorescence measured immediately before Fm′. Fm′ equals the maximum chlorophyll fluorescence when all reaction centers of photosystem II are closed due to the saturation light impulse of the instrument. PAM measurements were conducted with two Teaching‐PAMs (Gademann Instruments, Würzburg, Germany) in situ before, during and after a representative short fog event under ambient light. Eight replicates of each of the two terricolous lichen species next to the climate station as well as eight replicates of each of the two epiphytic lichens (Appendix Table A1) that were growing attached to cacti in close vicinity were measured at intervals of 3–5 min under foggy conditions. In the laboratory, an additional eight replicates of each lichen were submersed in water for 20 min to reach a fully hydrated state in the dark and measured again. Field measurements were calculated to their corresponding yield in a completely hydrated status and are therefore given in percent. Photosynthetic yield values can strongly vary between lichen species and are shown in percent to their maximum yield in order to compare the four lichen species (see Appendix Table A6 for original values). Yield values lower than 0.2 were treated as not active (Leisner, Bilger, & Lange, 1996). Temperature was recorded using the same instruments simultaneously with the PAM measurements.

### 2.10 Statistics

Statistics for LSP and LCP were calculated using the software Statistica (Version 9.1; StatSoft Inc. 2010). The data were tested for normal distribution using a Shapiro–Wilk test. After all data were found to be normally distributed and homogeneity of variances was verified using Levene test a one‐way ANOVA followed by Tukey post hoc test was used to detect differences between groups. Unless otherwise noted, significant differences refer to *p ≤ *0.05.

### 2.11 Protection of human subjects and animals

The protocol and procedures employed were reviewed and approved by an appropriate institutional review committee.

## 3 RESULTS

### 3.1 Climate

**Figure 1:**
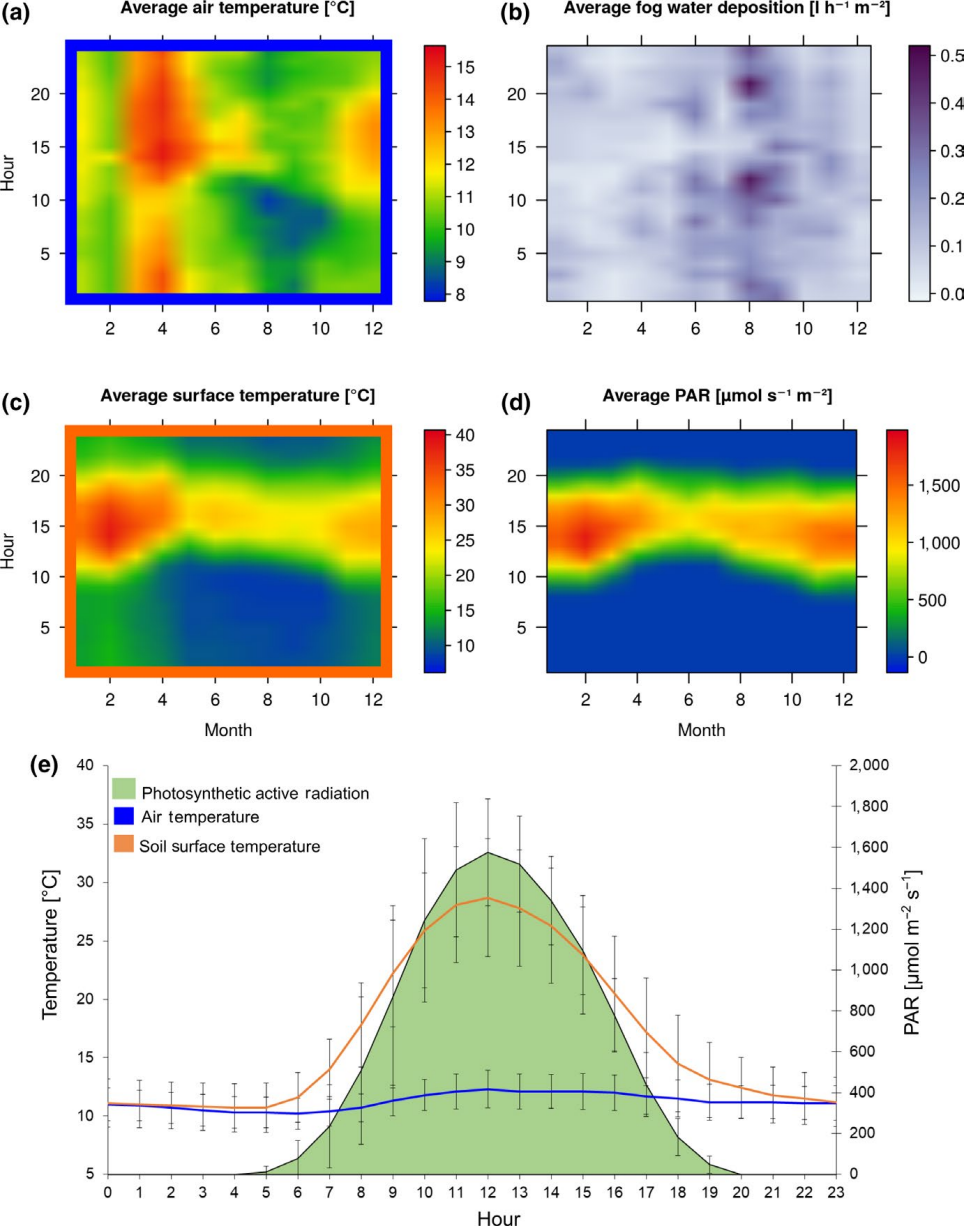
Climate recording from 2017. Given are the air temperature (a), fog water deposition (b), soil surface temperature (c) and PAR (photosynthetic active radiation) (d) as averages across the months of 2017 and the daytime. (e) Represents an average daily course of air and soil surface temperature and PAR during 2017 with standard deviations

Soil surface temperature (Figure 1a) and air temperature (Figure 1c) as well as PAR (Figure 1d) were highest during noon throughout the year. During summer (February–April) maximum temperatures of 40°C at surface level and 15°C in the air were recorded. Highest temperatures during winter (July–September) varied between 22°C at surface level and 11°C in the air. While soil surface temperature varied strongly during the day a constant air temperature of 12°C was recorded, (Figure 1e). PAR exceeded 2000 µmol m^−2^ s^−1^ at noon in summer time (Figure 1d). Fog water interception reached its maximum before noon during the winter period exceeding 490 ml h^−1^ m^−2^ (Figure 1b).

### 3.2 Phylogeny

**Figure 2:**
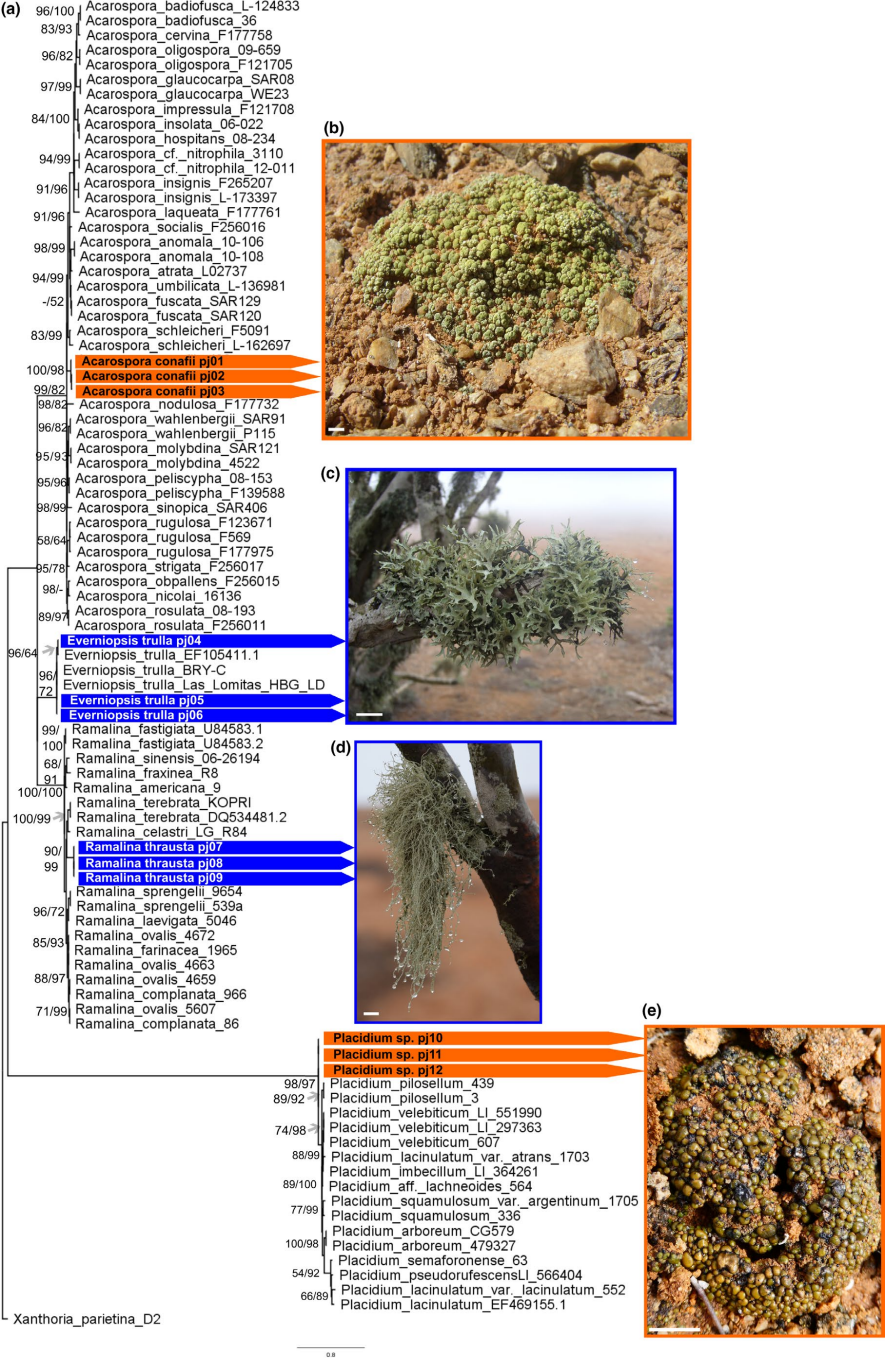
Phylogenetic tree of the lichen mycobionts rooted to *Xanthoria parietina* KJ027708.1 and micrographs. (a) Concatenated maximum‐likelihood tree (ITS + RBP1) of the mycobionts. Numbers at nodes represent first the ML bootstrap support (values ≥ 50%) and second the posterior probabilities from the Bayesian analysis (values ≥ 50%). The scale indicates the numbers of substitutions per site and generated sequences are connected by color code to the corresponding micrographs (orange = terricolous lichens; blue = epiphytic lichens). Micrographs of the terricolous *Acarospora conafii* is given in (b), *Everniopsis trulla* in (c), *Ramalina thrausta* in (d) and *Placidium* sp. in (e). Scale bar for images is 1 cm

The mycobiont phylogeny revealed that three of the newly generated sequence groups highly support the other *Placidium* species. Unfortunately, we were not able to assign them to species level because they are polyphyletic (Figure 2a,e). The sequences of the three replicates were identical to each other. The sampled *Acarospora* species joined an *Acarospora* clade (Figure 2a,b) but revealed low similarity (87%–94%) to other *Acarospora* sequences at species level. The sequences of *Everniopsis trulla* (Ach.) Nyl. from this study fall within a clade with other sequences of this species which originated from the study site vicinity (Figure 2a,c). The sequenced *Ramalina* species clustered with publicly available sequences of other *Ramalina* species but could not be assigned to any known species based on the obtained sequences (Figure 2a,d).

**Figure 3:**
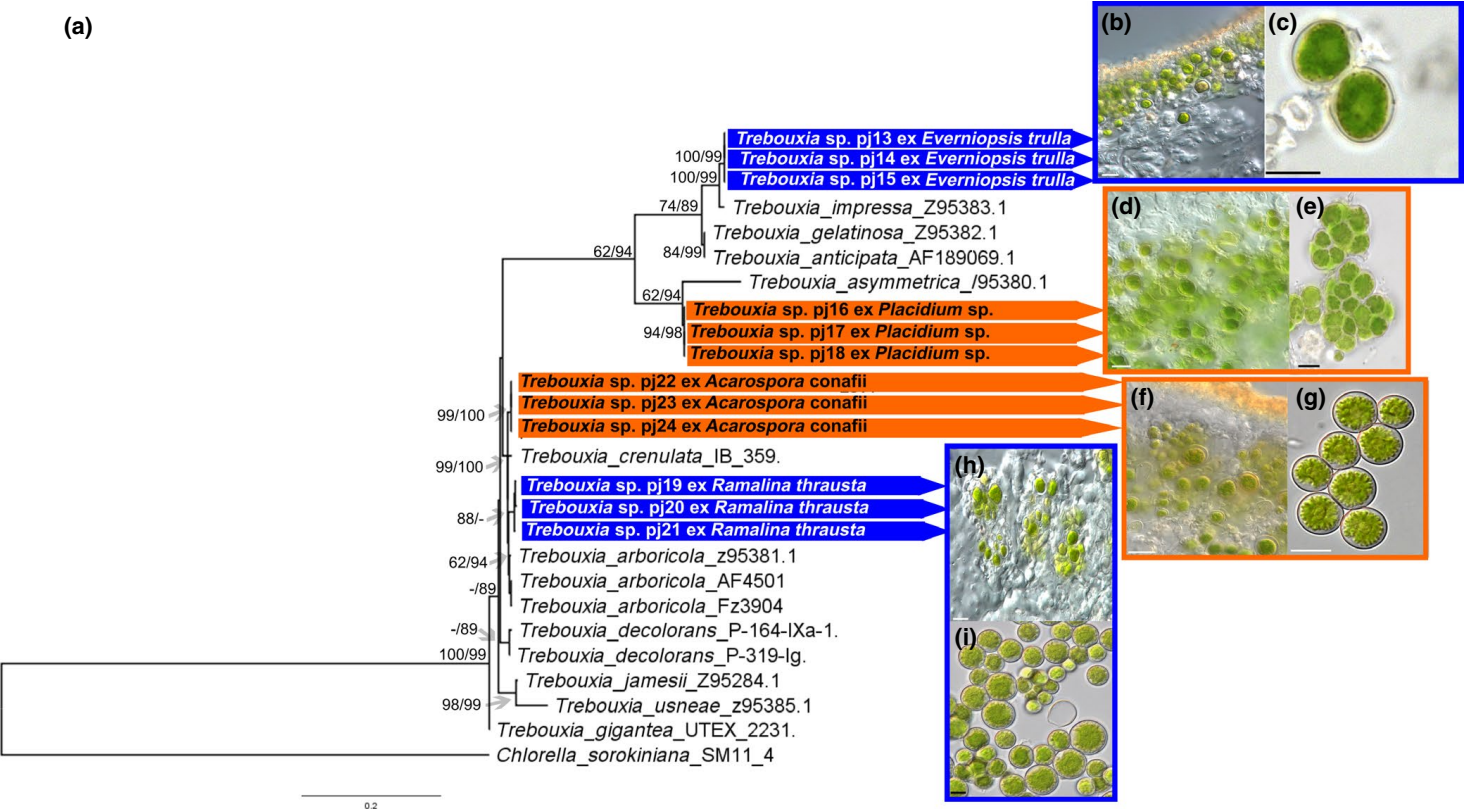
Phylogenetic tree of the lichen photobionts rooted to *Chlorella sorokiniana* KX495084.1 and micrographs. (a) Concatenated maximum‐likelihood tree (18S rDNA; 26S rDNA; rbcL) of the *Trebouxia* photobionts. Numbers at nodes represent first the ML bootstrap support (values ≥ 50%) and second the posterior probabilities from the Bayesian analysis (values ≥ 50%). The scale indicates the numbers of substitutions per site and generated sequences are connected by color code to the corresponding micrographs (orange = terricolous lichens; blue = epiphytic lichens). The micrographs show thin sections of the lichens with their photobionts (b, d, f, and h) and images taken from the photobionts in culture (c, e, g, and i) with a scale bar of 10 μm. (b and c) Show the photobionts of *Everniopsis trulla* (*Trebouxia impressa* clade), (d and e) of *Placidium* sp. (*T. asymmetrica* clade), (f and g) *Acarospora conafii* and *Ramalina thrausta* (h and i) (both *T. arboricola* clade)

The sequences of the photobionts from *Placidium* sp. were highly similar to *Trebouxia asymmetrica* Friedl & Gärtner, whereas the green algal photobiont of *E. trulla* joined the *Trebouxia impressa* clade and the *Acarospora* and *Ramalina* photobionts were highly similar to each other and to *Trebouxia arboricola* Puymaly but formed their own clades (Figure 3).

**Figure 4:**
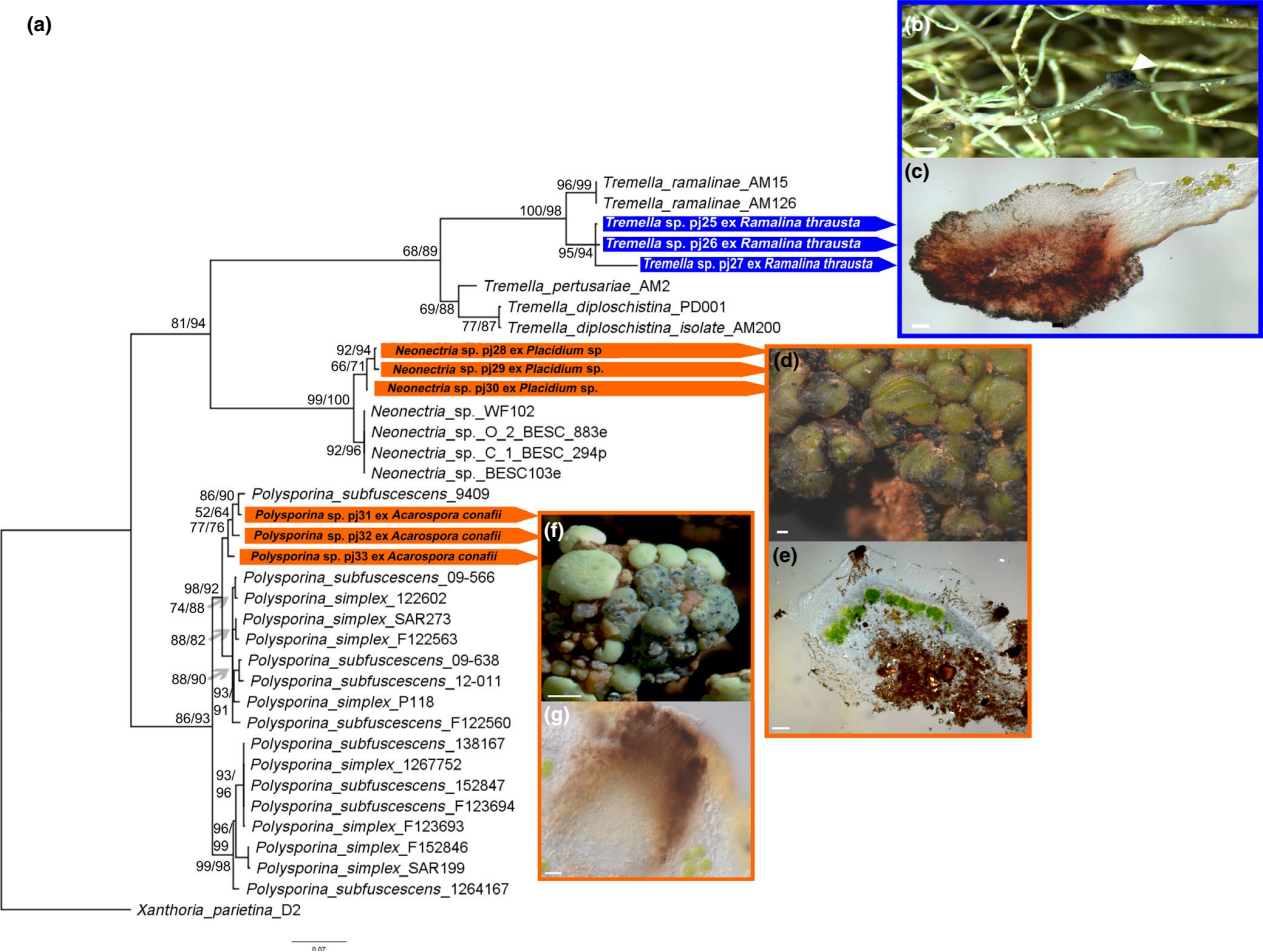
Phylogenetic tree of the lichenicolous fungi rooted to *Xanthoria parietina* KJ027708.1 and micrographs. (a) ITS maximum‐likelihood tree of the photobionts. Numbers at nodes represent the ML bootstrap support (values ≥ 50%) and second the posterior probabilities from the Bayesian analysis (values ≥ 50%). The scale indicates the numbers of substitutions per site and generated sequences are connected by color code to the corresponding micrographs (orange = terricolous lichens; blue = epiphytic lichens). Micrographs show the gall of *Tremella* sp. attached to *R. thrausta* (white triangle, b) and as thin section of the same gall (c). *Neonectria* sp. is shown on top of the thallus of *Placidium* sp. (d) and within the epinecral layer of the lichen (e). The infected thalli of *Acarospora conafii* are shown in (f) and *Polysporina* sp. within the pycnidia of the lichen in (g)

The *Ramalina* species was found to be affiliated with a black gall forming lichenicolous fungi of the genus *Tremella* (Figure 4a–c) that was highly related to *T. ramalinae* Diederich (98%) but formed a distinct cluster. The epinecral layer of the *Placidium* species was strongly colonized by a member of the genus of *Neonectria* (Figure 4a,d,e) which is a saprophytic fungus that only grew within the very top layer of the lichen, not penetrating the pigment layer. The crustose *Acarospora* species was strongly affiliated with a *Polysporina* species (Figure 3a,f,g) that clustered with a separated *Polysporina subfuscescens* clade with high similarity (98%) as the lichenicolous fungi.

### 3.3 Morphology

**Figure 5:**
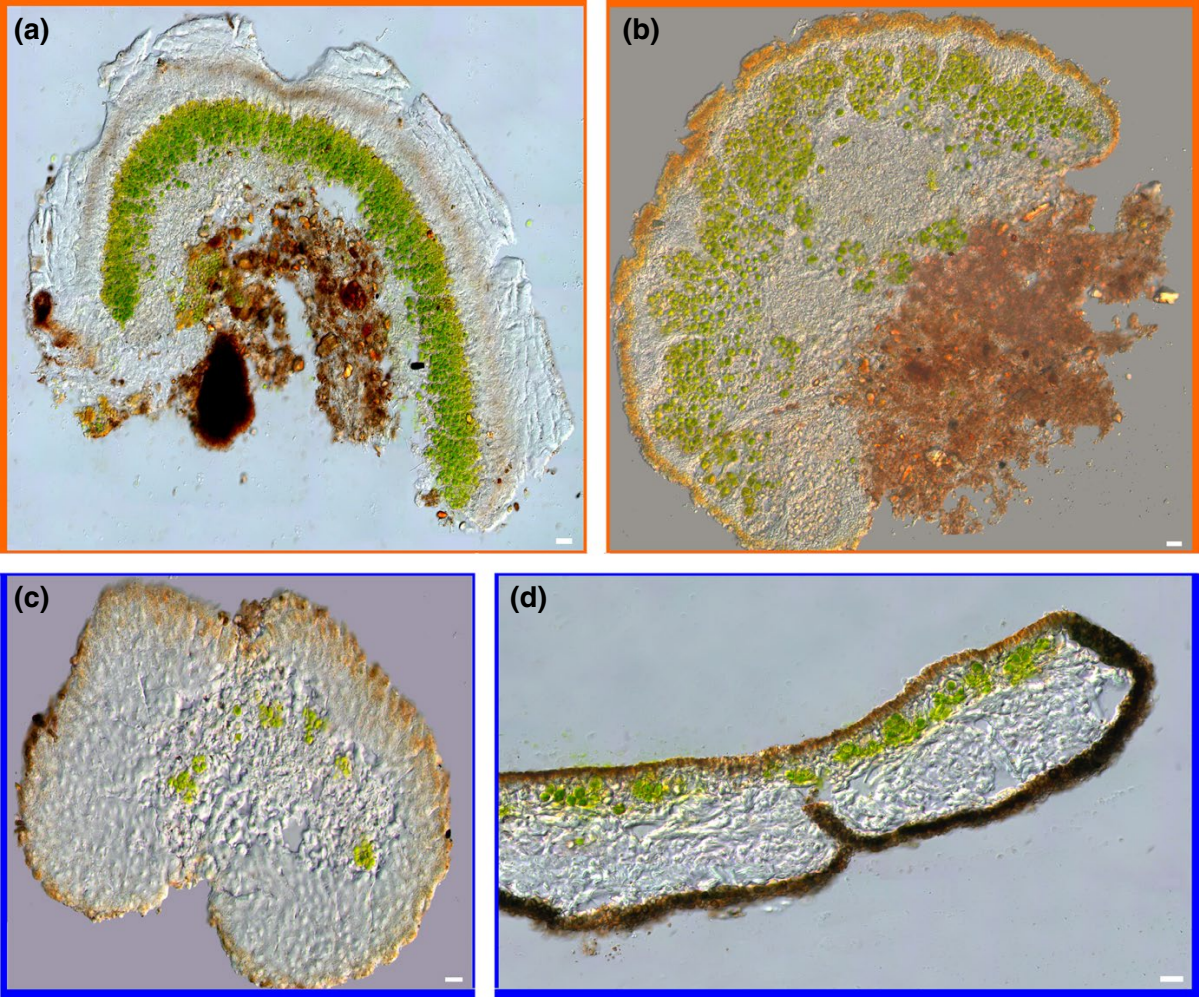
Microscopic cross‐sections of the investigated lichens. (a) Terricolous *Acarospora conafii*, (b) terricolous *Placidium* sp., both with vertical algal and fungal stacks. (c) Epiphytic *Ramalina thrausta* with photobiont nests and the epiphyte *Everniopsis trulla* with a horizontal photobiont layer (d). Scale bar represents 10 μm, each

The *Acarospora* species showed a morphology that did not fit to any of the described species of the genus and contained a photobiont layer that was arranged in vertical algal stacks that were sometimes discontinued by vertical fungal stacks (Figure 5b). Therefore it has been deemed a new species and was named *A. conafii* which was supported by the phylogenetic placement.

Similar to *A. conafii*, *Placidium* also showed the structure of algal and fungal stacks. The morphological features such as a prominent epinecral layer (Figure 5a) as well as the presence of two celled spores did not reflect any described species of the genus, nor did those of highly related genera such as *Heteroplacidium* or *Placidiopsis*. For these reasons the definite identity of *Placidium* sp. remains unresolved until further phylogenetic investigations will address this.

The morphological features of the investigated *Ramalina* species resembled those of *Ramalina thrausta* (Ach.) Nyl. and showed a nested orientation of the photobionts (Figure 5c).

A detailed description of the new species *A. conafii* is given below.

### 3.4 Species descriptions

***A. conafii* spec. nov. P. Jung et B. Büdel**

MycoBank No.: (830,228).

**Type**: Chile, Region de Atacama (III), National Park Pan de Azúcar, top of Coastal Cordillera, close to the coast in sites with fog events, 740 m. a.s.l., terricolous on quarzitic substrate, 24.vii.2017, P. Jung (Holotype‐HBG 024,635, isotype‐CONC 187,495).

**Thallus:** crustose to aerolate‐verrucose structure, pale yellow‐greenish to intensively light green. Areoles 0.25–1 mm in diameter, roundish to angular, convex; sometimes with crystals of gypsum on top of the upper cortex. Thallus morphology can be variable, some show warts on the upper cortex, others have more flexuouse than verrucose thalli. Upper cortex 40–110 µm thick, with the first 10–50 µm being pigmented; hyphae roundish, mucilaginous and translucent. Algal layer 40–70 µm thick with photobionts arranged in algal stacks; photobiont is the green alga *Trebouxia* sp., cells 8.5 µm in diameter. Medulla continuously present, about 120 µm thick, interspersed by crystals from the substratum, no further differentiation of hyphae. Lower cortex missing; thalli attached to the substratum without rhizines.

**Apothecia** not found.

**Pycnidia** Pycnidia globose, hyaline, 250–300 µm from bottom to top, 150 µm wide, surrounded by algae; walls hyaline, about 20 µm thick, prosoplectenchymatous with periclinally arranged hyphae; cavity convoluted. Pycnidiospores hyaline, long‐ellipsoid, 2.0–3.5 × 1.0–1.2 µm; convex wart like ring on top of the cortex around the ostiolum.

**Reactions**: K‐, C‐, KC‐, P‐

**Etymology:** The species is named after CONAF, Corporación Nacional Forestal, which is a Chilean private, nonprofit organization, through which the Chilean state contributes to the development and sustainable management of the country's forest resources.

**Remarks**: The species was found in close vicinity to *Caloplaca santessoniana* ad int., *Placidium* sp. and *Lecanora* sp. Compared to known Acarospora species this species is somehow similar to *A. gypsi‐deserti* Cl. Roux & V. Wirth described from the Namib Desert but our species differs by its bright yellow‐ greenish colour, smaller squamules and a convex ring around the ostiolum. Phylogenetic comparisons based on the internal transcribed spacer (ITS) and the large subunit of RNA polymerase II (RPB1) placed the species within the Acarospora clade. *Acarospora schleicheri* (Ach.) A. Massal. appeared as the most similar species on a genetic level.

**Ecology:** The species can easily be recognized in the field by its vivid yellow‐greenish color and grows as crusts that can cover several square meters. Strong parasitism on the lichen by the lichenicolous fungus (*Polysporina* sp.) has been observed in the field and is known to suppress the formation of apothecia. This parasite grows inside of the pycnidia in a way that makes the parasitized lichen species appear dark and matt.

### 3.5 Photosynthetic response to light

**Figure 6:**
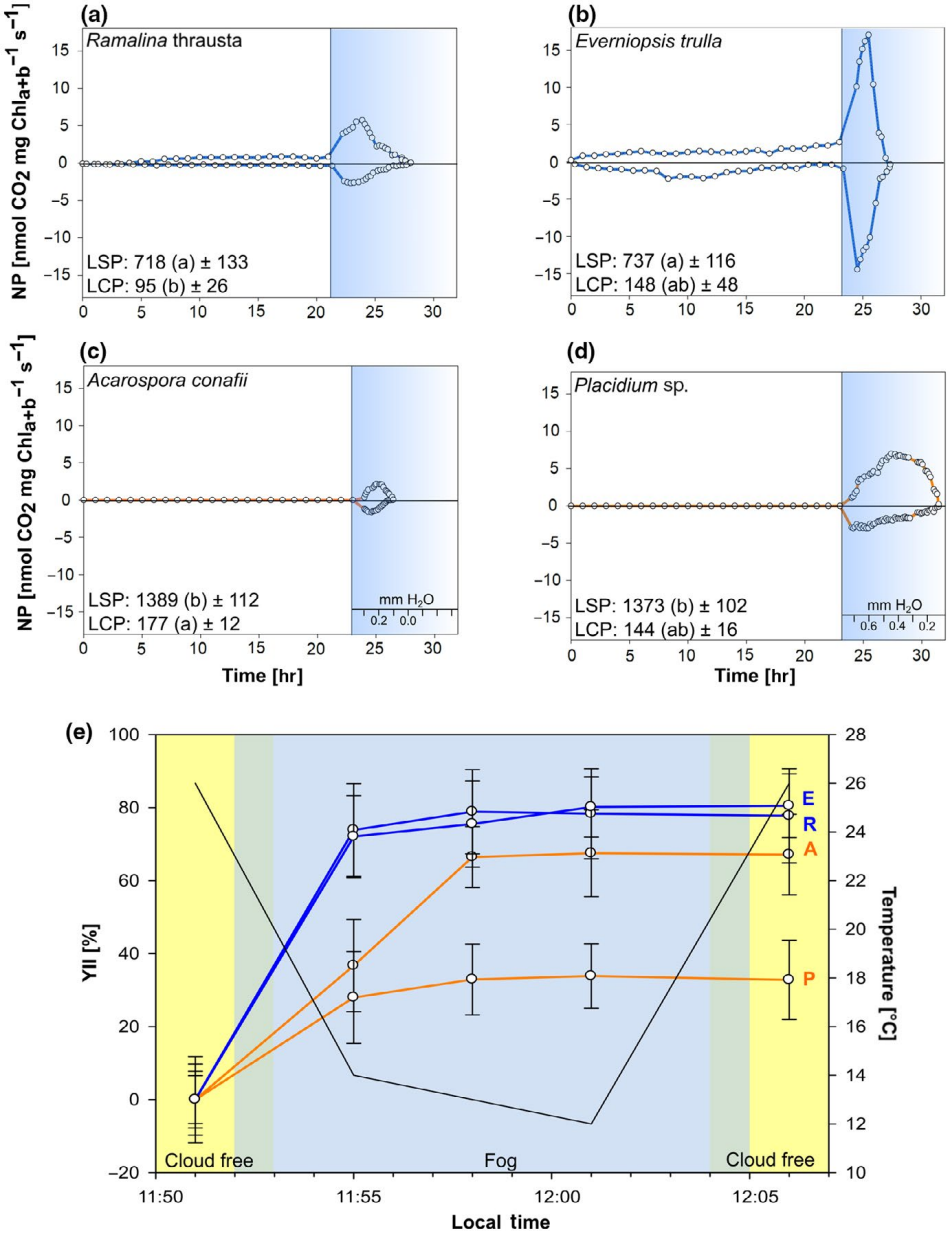
Ecophysiological measurements of lichens. Gas exchange measurements of the four lichens (a–d) during exposure of the dry thalli to 90% relative air humidity (white section) and after full hydration until they were completely dehydrated (blue section). Water content of the thalli is given in mm H_2_O as equivalent to precipitation for (c) and (d). LSP = light saturation point and LCP = light compensation point are given in μmol photons s^−1^m^2^ and standard deviation, different letters indicate significant differences between the LSP and LCP of the species, each (*n* = 5). PAM measurement before, during and after a fog event (e) shows the yield of photosystem II in percent, calculated down to the corresponding 100% yield reached after full hydration of the thalli and the temperature recorded during the measurements. E = *Everniopsis trulla*; R = *Ramalina thrausta*; A = *Acarospora conafii*; P = *Placidium* sp. (*n* = 8 for each species)

Photosynthetic patterns revealed high LSP of 1,326 (±113) µmol m^−2^ s^−1^ and 1,347 (±119) µmol m^−2^ s^−1^ for the two terricolous lichens *A. conafii* and *Placidium* sp. and significantly lower LSP of 737 (±116) µmol m^−2^ s^−1^ and 718 (±133) µmol m^−2^ s^−1^ for the epiphytes *E. trulla* and *R. thrausta* (Figure 6a–d) (ANOVA, *F*(3) = 25.56; *p* < 0.01;Tukey HSD test, MQ = 19,014 (FG) = 11;*p* < 0.01). *R. thrausta* reached positive net photosynthesis at significantly lower LCPs than *A. conafii* (ANOVA, *F*(3) = 5.44; *p* = 0.015; Tukey HSD test, MQ = 1,055; (FG) = 11; *p* = 0.012). The LCPs from all other species were similar to each other (between 95 and 177 µmol m^−2^ s^−1^).

### 3.6 Photosynthetic response to water

The two crustose lichens *A. conafii* and *Placidium* sp. did not react within 24 hr of exposure to air of 90% and 95% relative humidity (Figure 6a–d; white areas). The epiphytic lichen *R. thrausta* became active and showed positive net photosynthesis after 5 hr of exposure while *E. trulla* was reactivated already during the first hour after exposure. Reactivation patterns during an exposure to 95% relative air humidity showed a slightly higher respiration and net photosynthesis for *R. thrausta* and *E. trulla* than during 90% of exposure (data not shown).

During activation by liquid water the longest activity amplitude (7 hr of activity) was detected for *Placidium* sp. (Figure 6d; blue area) while the highest NP (18 nmol CO_2_ mg Chl_a+b_^−1^ s^−1^) and DR (13 nmol CO_2_ mg Chl_a+b_^−1^ s^−1^) were recorded for *E. trulla* (Figure 6b; blue area).

### 3.7 Chlorophyll fluorescence

The two terricolous lichen species *A. conafii* and *Placidium* sp. achieved 36.7% and 27.9%, respectively, of their maximum photosynthetic activity after the first 3 min of exposure to fog. During the further course of the experiment, *A. conafii* reached around 67% while *Placidium* sp. kept a maximum between 32% and 33% of its maximum yield.

The two epiphytes *E. trulla and R. thrausta* attached to cacti reached 73.9% and 72.1%, respectively, of their maximum photosynthetic yield after 3 min exposure to fog (Figure 6e). The values increased to 77.8% for *E. trulla* and to 80.5% for *R. thrausta* during further exposure to fog.

Temperature dropped during the fog event from 26°C to 12°C within 8 min. Experiments were repeated during comparable fog events and revealed identical results (data not shown).

## 4 DISCUSSION

### 4.1 Lichen phylogeny

The *Acarospora* sequences from GenBank utilized in this study represent the largest taxon sampling available with our addition of *A. conafii* as a new species (Westberg et al., 2015). Identifications within Verrucariaceae can be even more complicated due to a scarcity of discriminating morphological characters as well as a lack of phylogenetic data. These problems have also been highlighted and demonstrated for the mycobiont genera of *Placidium*, *Clavascidium*, *Heteroplacidium* and *Psora* by Prieto, Martínez, Aragón, Gueidan and Lutzoni (2012), Williams et al. (2017) and Moya et al. (2018) and prevent a clear assignment of the *Placidium* species investigated during this study. For these reasons, it is named *Placidium* sp. until closer phylogenetic investigations will clarify its identity based on a greater genetic data set.

To date some *Ramalina* species have been recorded for the coastal Atacama Desert that show a considerable morphological variation (Rundel, 1978; Santiago, Gonçalves, Gómez‐Silva, Galetovic, & Rosa, 2018) but this is the first record of *R. thrausta* for the southern hemisphere. Typical are the filamentous thin branches that are slightly and irregularly swollen, the small hooked shaped terminal soralia, the spot‐like to elongate pseudocyphellae and the presence of usnic acid only (Bowler, 1977). Apothecia are very rare in the Northern hemisphere and no information on the spore length is given. However, the specimen investigated here frequently does have apothecia and the spores are 9–16 × 2–4 µm. Interestingly there is a macaronesian species, *Ramalina chondrina*, that shares most morphological features with *R. thrausta*, but with apothecia containing spores of 14–17 × 5–6 µm and without hooked shaped soralia (Krog & Osthagen, 1980). It has been discussed previously whether both species would be synonyms or not but further taxonomic and phylogenetic studies of *R. thrausta* and *R. chondria* including the exemplars found in the Atacama Desert could solve this. However, *R. thrausta* was found to be the most dominant lichen extensively covering the tall, cylindrical cacti of *E. saint‐pieana* and in lower proportions also grew on *Euphorbia* shrubs. Together with other epiphytic lichens such as *Usnea* species, *R. thrausta* is part of the diet of Guanacos at Las Lomitas (personal observations) which has also been documented for Guanacos in northern parts of the desert where lichens comprise almost 70% of their diet (Raedeke & Simonetti, 1988). Hence, epiphytic lichens are likely to form the nutritional basis for higher trophic levels in the Atacama Desert, comparable to reindeer populations in the Arctic that feed on terricolous lichens (Cooper, Smith, & Wookey, 2001).

### 4.2 Associated lichenicolous fungi

At Las Lomitas, roughly 20% of the *A. conafii* population was found to be accompanied by a lichenicolous fungus highly related to *P. subfuscescens* (Nyl.) K. Knudsen & Kocourk (Figure 4a,f,g), which is a widely distributed, successful and aggressive parasite. This parasitic fungus invades the host's thalli through the ostioles of the pycnidia or perithecia and grows inside them (Figure 4f,g). It is known to suppress ascomata production of the host, to deplete the algal layer and finally destroy the host (Knudsen & Kocourkova, 2008). Besides *Acarospora, Caloplaca* species are also considered to be possible hosts. This was observed at the study site for *C. santessoniana* ad int. (Gaya et al., 2015), where up to 50% of the population was parasitized. Almost no specimens of *A. conafii* or *C. santessoniana ad int.* were found with apothecia, which might be caused by the strong parasitism (Knudsen & Kocourkova, 2008).

In contrast to the parasitic character of *P. subfuscescens*, a *Neonectria* species was found to persistently invade the epinecral layer of *Placidium* sp. (Figure 4a,d,e). According to the saprophytic character of some *Neonectria* species, the fungi stopped penetrating the lichen thallus at the pigmented layer, which was formed by living lichenized hyphae, spreading only within the epinecral layer composed of dead cells (Figure 4e).

*Ramalina thrausta* was moderately affiliated with a gall inducing *Tremella* sp. (Figure 4a–c) although lichenicolous species are among the most poorly known representatives of this genus (Millanes, Westberg, Wedin, & Diederich, 2012). *Tremella* species were reported from a wide range of lichen genera (Diederich et al. 2018), and they have been thought to be parasitic on the mycobiont (de los Rios, 2002; Millanes et al., 2012). Recently, Tuovinen at al. (2018) challenged their occurrence as uniformly mycoparasites by demonstrating that the hyphal stage of *Letharia* associated *Tremella* species are in close contact with algal cells. We cannot exclude interactions between the detected *Tremella* species and the photobionts of *R. thrausta* since we investigated only the gall stage of the lichenicolous fungus.

### 4.3 Structural adaptations

Interestingly, the photobiont layer of *A. conafii* and *Placidium* sp. is organized in vertical stacks that are separated by vertical channels of light‐transferring fungal hyphae known as algal and fungal stacks (Figure 5a,b). This feature has been described from other members of the same genus around the world as well as for *Diplotomma atacamae* Follmann another chlorolichen from the Atacama Desert, as a convergent evolutionary trait related to high insolation (Vondrak & Kubásek, 2013). However, according to results of the gas exchange experiments the presence of fungal and algal stacks did not extend the period of CO_2_ assimilation. PAR exceeded 2000 µmol photons s^−1^ m^−2^ during summer at the sampling site (Figure 1d), which was reflected in high LSP and LCP values of *A. conafii* and *Placidium* sp. Furthermore, the feature of fungal and algal stacks was initially thought to extend the period of CO_2_ assimilation but could not be supported by the authors nor within this study during gas exchange experiments.

In terms of morphology and ecophysiology the prominent epinecral layer of *Placidium* sp. may have played a crucial role since it was built from hyaline hyphae surrounded by a gelatinous matrix (Figure 5a). Hence, we assumed that the epinecral layer of *Placidium* sp. provided water to the photobiont layer underneath, increasing the duration of activity of the green algal cells. Acclimation of the epinecral layer to increased irradiation within a single species was observed for the cyanolichens *Peltigera rufescens* (Weiss) Humb. and *P. praetextata* (Sommerf.) Zopf (Dietz, Büdel, Lange, & Bilger, 2000). Additionally, the chlorolichen *Psora decipiens* (Hedw.) Hoffm. which also belongs to Verrucariaceae, shows an adaption towards xeric stress in a similar way to *Placidium* sp. by increasing the ability to take up water from the environment (Colesie et al., 2017). Morphological adaptation patterns were also found within the epiphytic lichens. For example, *R. thrausta* has a relatively thick cortex that was assumed to be a characteristic adaptation of fog zone lichens. A thick cortex may prevent mechanical damage and desiccation by wind, as has been shown especially for *Ramalina* species (Rundel & Bowler, 1974).

### 4.4 Ecophysiology

Surprisingly, both terricolous lichen species could not be reactivated by exposure to high relative air humidity (90%, Figure 6c,d or 95% data not shown) although this was long thought to be a common reactivation pattern in chlorolichens (Lange et al., 1986). Our results fit into a more recent understanding of lichen physiology, showing that other terricolous chlorolichens such as *Acarospora gwynnii* C. W. Dodge & E. D. Rudolph from very dry areas of the Darwin Mountains in Antarctica or *Teloschistes lacunosus* (Rupr.) Savicz from the Tabernas Drylands in Spain are unable to be reactivated by high air humidity between 98% and 100% alone (Colesie et al., 2014; Del Prado & Sancho, 2007). High relative air humidity is present at the Coastal Cordillera of the Atacama Desert due to the close vicinity to the Pacific Ocean even under cloud free conditions ranging from 80% to 85% at night and from 60% to 70% during daylight (Rundel et al., 1996). However, for most chlorolichens’ air humidity levels between 90% and 100% were shown to be sufficient to activate photosynthesis. The present levels of air humidity are likely rarely sufficient to activate lichen photosynthesis at Las Lomitas. Even a lower humidity is probably present at ground level due to higher soil surface temperatures and thus terricolous lichens of this environment could lack an adaptation mechanism allowing for reactivation by water vapor. In contrast, Del Prado and Sancho (2007) showed that *T. lacunosus* did not show photosynthetic activity although it was frequently exposed to relative air humidity ranging between 85% and 100% even at the pediment. They concluded that this is a long‐term effect within soil crust lichens from very dry environments where high atmospheric humidity and frequent maritime fogs are extremely favorable for lichen growth.

In contrast to the terricolous lichens, both epiphytes were able to be reactivated by high air humidity. This is especially interesting since the epiphytic *R. thrausta* and the terricolous *A. conafii* shared green algal photobionts that were highly similar to *T. arboricola.* This demonstrates that the ability to use high air humidity for photosynthetic activity may be mainly controlled by the mycobiont.

Fog provided as microscopic droplets of liquid water activated *A. conafii and Placidium* sp. within 3 min by up to 36.7% and 27.9% of their maximum activity, respectively. This suggests a fast photosynthetic reaction to even short fog events especially if compared to lichen fields from the Negev Desert which were dominated by the terricolous chlorolichens *Gyalolechia fulgens* (Sw.) Søchting, Frodén & Arup, *Squamarina cartilaginea* (With.) P. James and others where fog was not sufficient to activate photosynthesis (Veste, Littmann, Friedrich, & Breckle, 2001). During fog events *Placidium* sp. did not exceed 33% of its maximum activity as detected by PAM measurements (Figure 6e), suggesting that short fog events and water vapor are not sufficient to saturate the thallus of this lichen and its epinecral layer. This could be caused by a hydrophobic character of the thick epinecral layer as reported previously for various lichens (Lakatos, Rascher, & Büdel, 2006). On the other site, during gas exchange measurements *Placidium* sp. showed a longer duration of photosynthesis compared to *A. conafii* (Figure 6), which was probably caused by the increased water holding capacity of the well‐developed epinecral layer. Liquid water of more than 0.6 mm even caused photosynthetic depression of NP during the gas exchange measurements (Figure 6d). In addition, *Placidium* sp. had an optimum water content of 0.3–0.6 mm which was more than twice as high as for *A. conafii* (0.2 mm), probably caused by the epinecral layer. This is comparable to a study conducted by Büdel, Vivas, and Lange (2013), who found that the optimum water content of *Placidium squamulosum* from the Sonoran Desert ranged from 0.5 to 0.7 mm. Terricolous lichens from arid lands of Utah such as *Psora cerebriformis* W. A. Weber and *Diploschistes diacapsis* (Ach.) Lumbsch showed optimal NP rates during comparable measurements by Lange et al. 1997 at 17°C of 0.8 and >1 mm, respectively, demonstrating that *Placidium* sp. and especially *A. conafii* are adapted to very low amounts of water coming from fog and dew.

High LSP and LCP values of all four lichens were considered as typical for lichens from areas with high light intensities. The obtained values are comparable to those reported for e.g., the terricolous lichens *D. diacapsis*, *P. cerebriformis* and *Squamarina lentigera* (Weber) Poelt from arid lands of Utah (Lange et al. 1997) or two morphospecies of the epiphytic *Ramalina menziesii* Taylor from a coastal and an inland habitat in central California (Matthes‐Sears, Nash, & Larson, 1987).

The epiphytic species *R. thrausta* was found to be efficient in fog water interception leading to dripping water and thus might improve host plant water use by microenvironmental modification as it has been demonstrated for *Ramalina* species from the same study site (Stanton et al., 2014). Especially during the winter season oversaturation of the epiphytic lichens can be observed at Las Lomitas when fog depositions reach up to 490 ml h^−1^ m^−2^ (Figure 1b). In addition to heavy fog, also short fog events were sufficient for *R. thrausta* and also *E. trulla* which reached and sustained 80% of their total photosynthetic yield after 3 min (Figure 6e). In 1983, Lange and Redon showed that *E. trulla* and other *Ramalina* species growing as epiphytes in the fog oasis of Fray Jorge in the South of the Atacama Desert could thrive even under an extremely low water potential of −251 bar, highlighting their adaptions towards arid conditions. They could also demonstrate that high amounts of water vapor at Fray Jorge were found to saturate the lichen thalli during night, leading to efficient NP during the early morning hours (Lange & Redon, 1983).

## 5 CONCLUSION

The lichen community with terricolous and epiphytic chlorolichens from the fog oasis Las Lomitas within the National Park Pan de Azúcar in the Atacama Desert showed morphological and ecophysiological features that enabled them to cope with the present climatic and environmental conditions. It can be assumed that morphological features such as algal and fungal stacks or the swelling capacity of the epinecral layer of the two terricolous lichens are adaptations that enable them to thrive in this area of the Atacama Desert while ecophysiological traits such as a fast reactivation by high air humidity and fog were part of the success of the epiphytic lichens. Besides this, a high photobiont diversity of different *Trebouxia* species as well as various lichenicolous and saprophytic fungi such as *Tremella* sp., *Neonectria* sp. and *Polysporina* sp. demonstrated the hidden microbial diversity within the Atacama Desert. It remains speculative if the described *Acacrospora conafii* is endemic to this area or if it is restricted to other fog oases in the Atacama Desert or elsewhere.

## CONFLICT OF INTERESTS

The authors declare that they have no conflict of interest.

## AUTHOR CONTRIBUTIONS

PJ designed the study, conducted measurements and prepared the manuscript, DE sequenced the mycobiont, photobiont and parasites, MS prepared the micrographs, KB conducted field measurements, LW and CC analyzed the gas exchange patterns, LL, JB and SA recorded and analyzed the climatic data, PC helped during the identification of the lichens, LBW guided this work and BB designed the study and guided this work. All authors commented on the manuscript.

## ETHICS STATEMENT

The protocol and procedures employed were reviewed and approved by an appropriate institutional review committee in collaboration with the Chilean collaboration partners of the Corporación Nacional Forestal (CONAF).

## Data Availability

Generated sequences can be found under the project number PRJEB31788 at NCBI GenBank and all alignments are deposited at dryad https://doi.org/10.5061/dryad.jc06126.

## APPENDIX 1

**Table A1:** Description of experimental design showing the number of replicates that were used for each experiment with the GPS coordinates from the sampling location or the location where the experiment was conducted. All samples were collected from the National Park Pan de Azúcar, Atacama Desert, Chile

Experiment	Lichen Species	*n*	GPS coordinates (latS/longW)	m above sea level
PAM	*Acarospora conafii*	8	25; 59;04 70;36;56	769
PAM	*Placidium* sp.	8	25; 59;04 70;36;56	769
PAM	*Everniopsis trulla*	8	25;59;00 70;36;58	768
PAM	*Ramalina thrausta*	8	25;59;00 70;36;58	768
Eco‐physiology: response to light	*A. conafii*	5	25;59;00 70;36;58	757
			25;59;03 70;36;57	761
			26;00;47 70;35;58	874
			25;59;00 70;36;58	681
			25;59;02 70;36;18	762
Eco‐physiology: response to light	*Placidium* sp.	5	26;00;31 70;36;23	763
			25;58;59 70;36;54	715
			26;01;39 70;34;04	693
			25;59;00 70;36;58	721
			25;59;00 70;36;58	842
Eco‐physiology: response to light	*E. trulla*	5	25;59;00 70;36;58	753
			25;59;02 70;36;18	836
			26;00;23 70;36;19	693
			26;00;47 70;35;58	833
			25;59;02 70;36;55	763
Eco‐physiology: response to light	*R. thrausta*	5	25;59;00 70;36;58	753
			25;59;02 70;36;18	836
			26;00;23 70;36;19	693
			26;00;47 70;35;58	833
			25;59;02 70;36;55	763
Eco‐physiology: response to water	*A. conafii*	5	Same as above	
Eco‐physiology: response to water	*Placidium* sp.	5	Same as above	
Eco‐physiology: response to water	*E. trulla*	5	Same as above	
Eco‐physiology: response to water	*R. thrausta*	5	Same as above	
Eco‐physiology: exposure to high air humidity	*A. conafii*	5	Same as above	
Eco‐physiology: exposure to high air humidity	*Placidium* sp.	5	Same as above	
Eco‐physiology: exposure to high air humidity	*E. trulla*	5	Same as above	
Eco‐physiology: exposure to high air humidity	*R. thrausta*	5	Same as above	

**Table A2:** Lichen replicates used for DNA extraction, amplification and sequencing with their additional GenBank accession numbers. All samples were collected from the National Park Pan de Azúcar, Atacama Desert, Chile

Sample ID	Origin/GPS coordinates (latS/longW)	Marker	GB Accession
*Acarospora conafii* pj01	25; 59;04 70;36;56	nuITS	ERZ868389
*Acarospora conafii* pj02	25;59;00 70;36;58	nuITS	ERZ868424
*Acarospora conafii* pj03	26;00;31 70;36;23	nuITS	ERZ868445
*Everniopsis trulla* pj04	25; 59;04 70;36;56	nuITS	ERZ827871
*Everniopsis trulla* pj05	25;59;00 70;36;58	nuITS	ERZ827872
*Everniopsis trulla* pj06	26;00;23 70;36;19	nuITS	ERZ827874
*Acarospora conafii* pj01	see above	RPB1	ERZ868473
*Acarospora conafii* pj02	see above	RPB1	ERZ868489
*Acarospora conafii* pj03	see above	RPB1	ERZ868498
*Everniopsis trulla* pj04	see above	RPB1	ERZ827875
*Everniopsis trulla* pj05	see above	RPB1	ERZ868648
*Everniopsis trulla* pj06	see above	RPB1	ERZ868658
*Ramalina thrausta* pj07	25;59;00 70;36;58	nuITS	ERZ868517
*Ramalina thrausta* pj08	25;59;00 70;36;58	nuITS	ERZ868529
*Ramalina thrausta* pj09	25;59;00 70;36;58	nuITS	ERZ868539
*Placidium* sp. pj10	25;59;03 70;36;57	nuITS	ERZ868597
*Placidium* sp. pj11	25;59;00 70;36;58	nuITS	ERZ868604
*Placidium* sp. pj12	25; 59;04 70;36;56	nuITS	ERZ868614
*Ramalina thrausta* pj07	see above	RPB1	ERZ868566
*Ramalina thrausta* pj08	see above	RPB1	ERZ868574
*Ramalina thrausta* pj09	see above	RPB1	ERZ868583
*Placidium* sp. pj10	see above	RPB1	ERZ868622
*Placidium* sp. pj11	see above	RPB1	ERZ868631
*Placidium* sp. pj12	see above	RPB1	ERZ868639
*Tremella* sp. pj25	from *Ramalina thrausta* pj07	nuITS	ERZ893792
*Tremella* sp. pj26	from *Ramalina thrausta* pj08	nuITS	ERZ893795
*Tremella* sp. pj27	from *Ramalina thrausta* pj09	nuITS	ERZ893798
*Neonectria* sp. pj28	from *Placidium* sp. pj10	nuITS	ERZ893959
*Neonectria* sp. pj29	from *Placidium* sp. pj11	nuITS	ERZ893962
*Neonectria* sp. pj30	from *Placidium* sp. pj12	nuITS	ERZ893965
*Polysporina* sp. pj31	from *Acarospora conafii* pj01	nuITS	ERZ893812
*Polysporina* sp. pj32	from *Acarospora conafii* pj02	nuITS	ERZ893814
*Polysporina* sp. pj33	from *Acarospora conafii* pj03	nuITS	ERZ893816
*Trebouxia* sp. pj13	from *Everniopsis trulla* pj07	26S rDNA	ERZ869956
*Trebouxia* sp. pj14	from *Everniopsis trulla* pj08	26S rDNA	ERZ869957
*Trebouxia* sp. pj15	from *Everniopsis trulla* pj09	26S rDNA	ERZ869958
*Trebouxia* sp. pj16	from *Placidium* sp. pj10	26S rDNA	ERZ894043
*Trebouxia* sp. pj17	from *Placidium* sp. pj11	26S rDNA	ERZ894044
*Trebouxia* sp. pj18	from *Placidium* sp. pj12	26S rDNA	ERZ894046
*Trebouxia* sp. pj19	from *Ramalina thrausta* pj07	26S rDNA	ERZ894006
*Trebouxia* sp. pj20	from *Ramalina thrausta* pj08	26S rDNA	ERZ894009
*Trebouxia* sp. pj21	from *Ramalina thrausta* pj09	26S rDNA	ERZ894029
*Trebouxia* sp. pj22	from *Acarospora conafii* pj01	26S rDNA	ERZ893972
*Trebouxia* sp. pj23	from *Acarospora conafii* pj02	26S rDNA	ERZ893976
*Trebouxia* sp. pj24	from *Acarospora conafii* pj03	26S rDNA	ERZ893978
*Trebouxia* sp. pj13	from *Everniopsis trulla* pj07	18S rDNA	ERZ893459
*Trebouxia* sp. pj14	from *Everniopsis trulla* pj08	18S rDNA	ERZ893480
*Trebouxia* sp. pj15	from *Everniopsis trulla* pj09	18S rDNA	ERZ893488
*Trebouxia* sp. pj16	from *Placidium* sp. pj10	18S rDNA	ERZ894048
*Trebouxia* sp. pj17	from *Placidium* sp. pj11	18S rDNA	ERZ894050
*Trebouxia* sp. pj18	from *Placidium* sp. pj12	18S rDNA	ERZ894052
*Trebouxia* sp. pj19	from *Ramalina thrausta* pj07	18S rDNA	ERZ894031
*Trebouxia* sp. pj20	from *Ramalina thrausta* pj08	18S rDNA	ERZ894033
*Trebouxia* sp. pj21	from *Ramalina thrausta* pj09	18S rDNA	ERZ894034
*Trebouxia* sp. pj22	from *Acarospora conafii* pj01	18S rDNA	ERZ893982
*Trebouxia* sp. pj23	from *Acarospora conafii* pj02	18S rDNA	ERZ893985
*Trebouxia* sp. pj24	from *Acarospora conafii* pj03	18S rDNA	ERZ893988
*Trebouxia* sp. pj13	from *Everniopsis trulla* pj07	rbcL	ERZ893581
*Trebouxia* sp. pj14	from *Everniopsis trulla* pj08	rbcL	ERZ893605
*Trebouxia* sp. pj15	from *Everniopsis trulla* pj09	rbcL	ERZ893622
*Trebouxia* sp. pj16	from *Placidium* sp. pj10	rbcL	ERZ894056
*Trebouxia* sp. pj17	from *Placidium* sp. pj11	rbcL	ERZ894058
*Trebouxia* sp. pj18	from *Placidium* sp. pj12	rbcL	ERZ894060
*Trebouxia* sp. pj19	from *Ramalina thrausta* pj07	rbcL	ERZ894036
*Trebouxia* sp. pj20	from *Ramalina thrausta* pj08	rbcL	ERZ894039
*Trebouxia* sp. pj21	from *Ramalina thrausta* pj09	rbcL	ERZ894042
*Trebouxia* sp. pj22	from *Acarospora conafii* pj01	rbcL	ERZ893993
*Trebouxia* sp. pj23	from *Acarospora conafii* pj02	rbcL	ERZ893998
*Trebouxia* sp. pj24	from *Acarospora conafii* pj03	rbcl	ERZ894003

**Table A3:** Publicly available sequences used for the alignments of the mycobiont

Species	Accession numbers	
	nuITS	RPB1
Placidium		
Placidium aboreum CG 579/AFTOL 2285	KY769559.1	EF689767.1
Placidium aboreum 479327	GU228968.1	GU229006.1
Placidium aff. lachneoides Prieto 564	GU228972.1	GU229005.1
Placidium imbecillium LI 364261	GU228979.1	GU229012.1
Placidium lacinulatum var. atrans 1703	GU228957.1	GU229019.1
Placidium lacinulatum var. lacinulatum Prieto 552	GU228960.1	GU229003.1
Placidum pillosellum 3	GU229013.1	GU229013.1
Placidium pillosellum Prieto 439	GU228968.1	GU229008.1
Placidium pseudorufescens LI 566404	GU228966.1	x
Placidium semaforense Prieto 63	GU228961.1	GU229002.1
Placidium squamulosum Prieto 336	GU228994.1	GU229009.1
Placidium squamulosum var. argentinium Prieto 1705	GU228991.1	GU229014.1
Placidium velebiticum LI 297363	GU228975.1	GU229007.1
Placidium velebiticum LI 551990	GU228978.1	x
Placidium velebiticum Prieto 664/607	GU228996.1	GU229022.1
Acarospora		
Acarospora anomala Westberg 10‐106	LN810758.1	x
Acarospora anomala Westberg 10‐108	LN810759.1	x
Acarospora atrata Arup L02737	LN810760.1	x
Acarospora badiofusca L‐124833	LN810762.1	x
Acarospora badiofusca Nordin Owe‐Larsson 36	LN810763.1	x
Acarospora cervina F177758	LN810764.1	x
Acarospora cf. nitrophila Westberg 12‐011	LN810786.1	x
Acarospora cf. nitrophila Westberg 3110	LN810787.1	x
Acarospora fuscata Westberg SAR120	LN810766.1	x
Acarospora fuscata Westberg SAR129	LN810767.1	x
Acarospora glaucocarpa Westberg SAR08	LN810768.1	x
Acarospora glaucocarpa Westberg WE23	LN810769.1	x
Acarospora hospitans Westberg 08‐234	LN810775.1	x
Acarospora impressula F121708	LN810776.1	x
Acarospora insignis F265207	LN890274.1	x
Acarospora insignis/L173397	LN890273.1	x
Acarospora insolata Westberg 06‐022	LN810777.1	x
Acarospora laqueata F177761/AFTOL 1007	LN810778.1	DQ782860.1
Acarospora molybdina 4522	AY853352.2	x
Acarospora molybdina Westberg SAR121	LN810738.1	x
Acarospora nicolai Morse 16136	LN810785.1	x
Acarospora nodulosa F177732	LN810789.1	x
Acarospora obpallens F256015	LN810790.1	x
Acarospora oligospora F121705	LN810791.1	x
Acarospora oligospora Westberg 09‐659	LN810792.1	x
Acarospora peliscypha Westberg 08‐153	LN810794.1	x
Acarospora peliscypha F139588	LN810793.1	x
Acarospora rosulata F256011	LN810796.1	x
Acarospora rosulata Westberg 08‐193	LN810797.1	x
Acarospora rugulosa F123671	LN810798.1	x
Acarospora rugulosa F569	DQ374147.1	x
Acarospora schleicheri F5091	AY853353.2	DQ782859.1
Acarospora schleicheri L‐162697	LN810801.1	DQ915591.1
Acarospora sinopica SAR406	DQ374148.1	x
Acarospora socialis F256016	LN810802.1	x
Acarospora strigata F256017	LN810805.1	x
Acarospora umbilicata L‐136981	LN810808.1	x
Acarospora wahlebergerii Westberg P115	LN810810.1	x
Acarospora wahlebergerii Westberg SAR91	LN810809.1	x
Everniopsis		
Everniopsis trulla	EF105411.1	EF105429.1
Everniopsis trulla Las Lomitas HBG LD	AY251419.1	x
Everniopsis trulla BRY‐C	KY859517.1	x
Ramalina		
Ramalina ovails 4663	KF583549.1	x
Ramalina americana 9	AF109235.1	x
Ramalina celastri LGR84	GU827295	x
Ramalina complanata AFTOL 86	x	DQ973059.1
Ramalina complanata AFTOL 966	x	DQ883782.1
Ramalina farinacea AFTOL 1965	x	KJ766831.1
Ramalina fastigata U84583.1	U84583.1	AY756422.1
Ramalina fastigata U84583.2	U84583.2	KJ766832.1
Ramalina fraxinea R8	KC960763.1	x
Ramalina laevigata 5046	KY171864.1	x
Ramalina ovalis 4659	KF583548.1	x
Ramalina ovalis 4672	KF583554.1	x
Ramalina ovalis 5607	KF583558.1	x
Ramalina sinensis 06‐26194	JF923611.1	x
Ramalina sprengelii 9654	KY171867.1	x
Ramalina sprengelii 536a	KY171868.1	x
Ramalina terebrata DQ534481.2	DQ534481.2	x
Ramalina terebrata KOPRI	EU161239.1	x
Root		
Xanthoria parietina	KT695329.1	JQ301734.1

**Table A4:** Publicly available sequences used for the alignments of the photobionts

Species	Accession numbers		
	26S rDNA (LR3)	18 S rDNA (Al1500af)	rbcL
Trebouxia impressa Z95383.1	Z95383.1	x	AB194849.1
Trebouxia gelantinosa Z95382.1	Z95382.1	x	AJ969640.1
Trebouxia anticipata AF189069.1	AF189069.1	x	AB194852.1
Trebouxia asymmetrica 95380.1	Z95380.1	Z21553.1	AB194860.1
Trebouxia jamesii Z95284.1	Z95284.1	x	AJ969663.1
Trebouxia usnea z95385.1	Z95385.1	x	x
Trebouxia decolorans P‐164‐Ixa‐1	AJ969563.1	AJ969562.1	AJ969660.1
Trebouxia decolorans P‐319‐lg	AJ970889.1	FJ705185.1	AJ969668.1
Trebouxia arboricola z95381.1	Z95381.1	JQ993785.1	AJ969669.1
Trebouxia arboricola AF4501	Z95381.1	JQ993784.1	AJ969670.1
Trebouxia arboricola Fz3904	Z95381.1	JQ993786.1	AJ969666.1
Trebouxia crenulata IB359	x	x	AJ969639.1
Trebouxia gigantea UTEX 2231	x	x	AB194855.1
Root			
Cholrella sorokiniana SM11 4	KM514858.1	KM291883.1	HM101339.1

**Table A5:** Publicly available sequences used for the alignments of the lichenicolous fungi

Species	Acession number nuITS
Tremella ramalinae AM15	JN053513.1
Tremella ramalinae AM126	JN043619.1
Tremella pertusaria AM2	JN043600.1
Tremella diploschistina PD001	JN790589.1
Tremella diploschsitina isolate AM200	JN790590.1
Neonectria sp. WF102	HQ130662.1
Neonectria sp. O 2 BESC 883e	KC007320.1
Neonectria sp. C 1 BESC 294p	KC007272.1
Neonectria sp. BESC 103e	KC007134.1
Polysporina subfuscecens 9490	LN810847.1
Polysporina subfuscecens 09‐566	LN810838.1
Polysporina simplex 122602	LN810819.1
Polysporina simplex SAR 273	LN810826.1
Polysporina simplex F122563	LN810823.1
Polysporina subfuscescens 09‐638	LN810837.1
Polysporina subfuscescens 12‐011	LN810842.1
Polysporina simplex P118	LN810825.1
Polysporina subfuscescens F123694	LN810833.1
Polysporina subfuscescens 152847	LN810845.1
Polysporina subfuscescens F122560	LN810830.1
Polysporina subfuscescens 138167	LN810831.1
Polysporina simplex 1267752	LN810827.1
Polysporina simplex F123693	LN810818.1
Polysporina simplex F152846	LN810832.1
Polysporina simplex SAR199	LN810821.1
Polysporina subfuscescens 1264167	LN810848.1
Root	
Xanthoria parietina	KT695329.1

**Table A6:** In situ pulse amplitude modulation (PAM) measurements. Given are the actual mean Yield values of photosystem II of four lichens and the standard deviations obtained during a fog event

	*Acarospora conafii*	*Placidium* sp.	*Everniopsis trulla*	*Ramalina thrausta*
Local Time	Yield II (mean) ± standard deviation			
11:51	0.0729 ± 0.0268	0.0529 ± 0.0168	0.1057 ± 0.0431	0.0978 ± 0.0642
11:55	0.2001 ± 0.0553	0.1801 ± 0.0353	0.4503 ± 0.0573	0.3812 ± 0.0771
11:58	0.3621 ± 0.0773	0.2121 ± 0.0673	0.4809 ± 0.0554	0.3996 ± 0.0632
12:01	0.3679 ± 0.0769	0.2181 ± 0.0673	0.4776 ± 0.0589	0.4241 ± 0.0690
12:06	0.3659 ± 0.0989	0.2111 ± 0.0673	0.4741 ± 0.0612	0.4259 ± 0.0671
Wet	0.5450 ± 0.0698	0.6441 ± 0.0592	0.6097 ± 0.0599	0.5290 ± 0.0612
